# Hepatitis E in England and Wales

**DOI:** 10.3201/eid1401.070307

**Published:** 2008-01

**Authors:** Hannah C. Lewis, Sophie Boisson, Samreen Ijaz, Kirsten Hewitt, Siew Lin Ngui, Elizabeth Boxall, Chong Gee Teo, Dilys Morgan

**Affiliations:** * Health Protection Agency Centre for Infections, London, United Kingdom; † West Midlands Public Health Laboratory, Birmingham, United Kingdom

**Keywords:** hepatitis E virus, epidemiology, zoonosis, dispatch

## Abstract

In 2005, 329 cases of hepatitis E virus infection were confirmed in England and Wales; 33 were confirmed indigenous infections, and a further 67 were estimated to be indigenous infections. Hepatitis E should be considered in the investigation of patients with hepatitis even if they have no history of travel.

Acute hepatitis caused by hepatitis E virus (HEV) has historically been considered an imported disease associated with travel to a disease-endemic area. However, an increasing number of sporadic hepatitis E cases not associated with travel have been reported from industrialized countries, including England and Wales (*1*).

Four HEV genotypes have been identified that correlate with the geographic origin of the virus (*2*). Genotype 1 is regularly identified with disease-hyperendemic areas such as Africa and southern Asia; genotype 2, with Mexico and West Africa; genotype 3, with industrialized countries such as North America, Europe, and Japan; and genotype 4, with eastern Asia and India.

The epidemiology of non–travel-associated hepatitis E is largely unknown. Although the main route of transmission in disease-hyperendemic areas is the consumption of fecally contaminated water, risk factors for HEV infection in non–disease-hyperendemic countries have included occupational exposure to pigs (*3*) and consumption of raw or undercooked pork products (*4*), shellfish (*5*), and venison (*6*).

HEV is endemic in pig populations worldwide (*7*), including England and Wales, where HEV genotype 3 is widespread in pigs, and subgenomic sequencing studies have shown a close relationship between pig and human HEV strains (*8*). Although zoonotic transmission from swine to humans appears plausible, the possibility of a reservoir common to both swine and humans cannot be excluded.

## The Study

The reference laboratory for hepatitis E at the Health Protection Agency’s Centre for Infections reported 17 cases of non–travel-associated hepatitis E in England and Wales from 1996 through 2003 (*5*). During 2004, that laboratory and the other hepatitis E reference laboratory in Birmingham received an increased number of samples for HEV testing with a corresponding increase in numbers of hepatitis E cases diagnosed by testing for HEV immunoglobulin (Ig) M and IgG by using ELISAs (Genelabs Technologies Inc, Redwood City, CA, USA). These observations led to this study, the aims of which were to describe the epidemiology of hepatitis E in England and Wales, estimate the number of non–travel-associated cases, and identify risk factors for HEV infection in indigenous cases.

**Figure 1:**
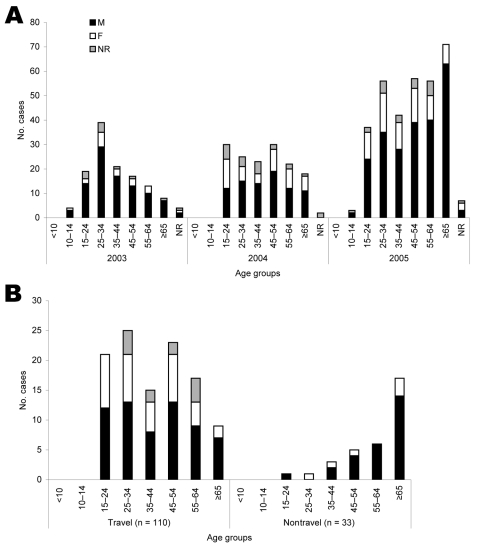
A) Age and sex distribution of acute hepatitis E patients, 2003–2005 (n = 604). B) Age, sex distribution, and travel associated with indigenous acute hepatitis E case-patients (n = 143). NR, not recorded.

During 2005, the reference laboratories confirmed 329 cases of acute hepatitis E infection. This number represented a substantial increase from 125 cases in 2003 and 150 in 2004. Information on the age groups and sex of all patients who received a diagnosis during 2003, 2004, and 2005 is shown in Figure 1. This figure shows a progressive increase in the number of acute cases of hepatitis E in older male patients over the period, and panel** B** demonstrates the overrepresentation of older men among those with established indigenous cases during 2005 compared with those who were known travelers.

**Figure 2:**
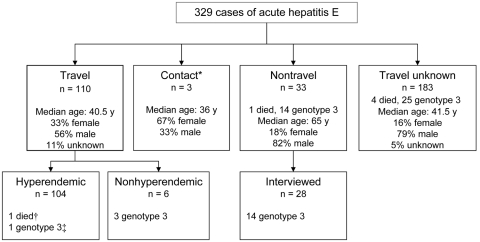
Details of cases of acute hepatitis E (HEV) infections, 2005. *Contact with hepatitis E patients who recently returned from hyperendemic countries; †24-year-old woman infected with HEV genotype 1; ‡45-year-old Caucasian man who traveled to Iraq.

The travel history of the 329 patients is summarized in Figure 2. For 102 (31%) patients, pre-illness travel information was recorded on the laboratory request form. Travel status was obtained for 44 additional patients through follow-up, and 33 patients who had not traveled outside the United Kingdom were considered to be indigenous case-patients. Twenty-three (70%) indigenous case-patients were >55 years of age, compared with 26 (24%) of 110 travel-associated case-patients; the median age was 65 years (interquartile range [IQR] 50–74 years) and 41 years (IQR 29–54) (p<0.0001) respectively. Thirty-two of the indigenous case-patients (97%) had Caucasian names (name classification was used as a proxy for ethnicity) compared with 21 (19%) of 110 travelers (p<0.001).

The number of indigenous HEV infections was estimated for those with an unknown travel history by fitting a logistical regression model that used Caucasian name and age as predictor variables. An estimated 67 (95% confidence interval 58–75) of the 176 patients (when the date of birth was known) in this group acquired their infection in England and Wales.

Twenty-eight indigenous case-patients were available for telephone interview in which a detailed structured questionnaire was used to identify potential risk factors over the 9-week period before illness. The Table summarizes the results obtained from responses. All patients were white, and 23 (82%) were male, with a median age of 65 years. Twenty (71%) were referred from southern England and Wales; a similar proportion of interviewed patients lived at inland or coastal addresses, and a high proportion of patients lived in densely populated urban areas. No common risk factors were identified apart from 17 (60%) owning pets.

**Table:** Main risk factors for cases of indigenous hepatitis E, England and Wales, 2005*

Risk factor	Yes/ possibly	No
Residence		
Coastal address (<20 miles from sea)	15	13
Urban area (>10,000 inhabitants)	27	1
Animal exposure		
Occupation involving animal exposure	1	27
Own pets	17	11
Live on/visited a farm	4	24
Food preferences (i.e., likely to have consumed in the 9 weeks before onset of illness)		
Pork	22	5
Raw/undercooked pork	0	27
Pig liver	7	19
Raw/undercooked pig liver	0	26
Venison	0	25
Chicken	24	2
Raw/undercooked chicken	0	27
Other meat (beef, lamb, turkey)	18	8
Raw/undercooked other meat	4	21
Fish	18	9
Shellfish	5	22
Food preparation		
Handle raw meat for cooking	13	15
Wash fruits	18	10
Wash vegetables	20	5
Drinking water supply		
Mains	21	6
Bottled water	15	13
River/stream or well	2	26
Recreational water exposure	5	23
Dinghy sailing	2	25
Fishing	4	24
Swimming (pool)	1	26

*n = 28; some respondents did not answer all questions, so not all cells add up to 28.

HEV RNA was detected in 14 of the 33 patients with indigenous hepatitis E, and all virus strains belonged to genotype 3. All but one of the genotype 3 nucleotide sequences clustered with previously described strains from the United Kingdom and Europe. Three distinct sequence groups were noted, but subsequent detailed analysis showed no correlation between these groups and geographic distribution or time, which indicated that a common source was unlikely.

## Conclusions

The number of hepatitis E cases diagnosed in England and Wales began increasing substantially after 2004. In 2005, acute hepatitis E was diagnosed in 329 patients; 33 of these definitely had acquired their infection in England and Wales, and a further 67 were estimated to have acquired their infection there. These figures may nevertheless be an underestimation since we also found that a high proportion of clinicians do not test for HEV unless the patient reports a recent history of travel to a disease-endemic area. This practice may be changing, and the progressive increase in the number of older patients with diagnoses of hepatitis E may reflect a greater awareness among clinicians. Local interest in hepatitis E may well explain the geographic distribution of indigenous cases.

The demographic features of patients who acquired their infection in England and Wales are striking. Most patients were Caucasian men >55 years of age. These findings corroborate other studies in the literature (*9*). Possible reasons for this high attack rate in older adults remain unclear. Men infected with HEV are more likely to access healthcare than women in both disease-hyperendemic and non–disease-hyperendemic countries (*5*,*10*). Some researchers have suggested that risk factors for disease may be linked to male occupational or societal roles (*2*). These unique demographic findings need to be emphasized to the medical community because older Caucasian men may not be perceived as being at risk by physicians.

The epidemiology of hepatitis E in non–disease-endemic countries remains poorly understood, and the results from seroprevalence studies vary greatly, which suggests that commercially available serologic tests may not be reliable for population surveys on genotype 3 infections. Despite in-depth telephone interviews, we did not identify a likely common source of infection, and genotyping data suggest multiple sources of exposure to HEV. We found that 60% of patients reported owning pets, a higher proportion than the proportion of animal-owning households in the United Kingdom for cats (25%) and dogs (21%) (*11*). Similar findings have been reported in the Netherlands (*12*). Although this difference might just reflect the fact that pet ownership is higher among older persons, a high prevalence of anti-HEV antibodies has been found among dogs in India (*13*) and cats in Japan (*14*), and a patient with hepatitis E from Japan owned a pet cat who was positive for antibodies to HEV (*15*), all of which indicate that domestic animals may be a potential reservoir for infection.

With the growing recognition of hepatitis E as an increasingly important zoonotic infection in England and Wales, HEV should be considered an etiologic agent of acute and fulminant hepatitis even in patients who report no travel history. We need to raise awareness among health professionals to consider hepatitis E when investigating non–travel-associated cases of hepatitis.
